# Rhabdomyolysis-Induced Resistant Hypercalcemia During the Recovery Phase of Acute Kidney Injury

**DOI:** 10.7759/cureus.108932

**Published:** 2026-05-15

**Authors:** Amit Das, Jorge Silva, Kana Miyata, John Edwards, Ezequiel Bellorin-Font

**Affiliations:** 1 Internal Medicine - Nephrology, Saint Louis University School of Medicine, St. Louis, USA

**Keywords:** acute kidney injury, dialysis, hypercalcemia, rhabdomyolysis, zoledronate

## Abstract

Rhabdomyolysis is a clinical syndrome characterized by skeletal muscle injury and the release of intracellular contents into the circulation. While early hypocalcemia is common, hypercalcemia during the recovery phase is a rare but serious complication. We present a case of a 36-year-old male who developed severe, refractory hypercalcemia following rhabdomyolysis-induced acute kidney injury. Despite standard treatment with loop diuretics, low-calcium hemodialysis, and calcitonin, the hypercalcemia persisted. In this report, we highlighted a rebound hypercalcemia occurring during recovery from rhabdomyolysis, a biphasic disturbance in calcium homeostasis, and emphasized the importance of careful monitoring and timely, judicious management.

## Introduction

Rhabdomyolysis is a clinical syndrome with a broad etiology, including trauma, ischemia, and drug toxicity, among others, and is characterized by injury and breakdown of skeletal muscle with the release of intracellular contents into the circulation [1,2]. This process results in local and systemic complications, comprising electrolyte disturbances, such as hyperkalemia, hyperphosphatemia, hypocalcemia, and hyperuricemia, as well as renal injury largely driven by myoglobin-mediated tubular toxicity, intrarenal crystal deposition, and associated glomerular damage [3,4]. An early hallmark of rhabdomyolysis is hypocalcemia, thought to result from rapid calcium influx into damaged muscle cells and calcium phosphate deposition within injured muscle. In contrast, hypercalcemia during the recovery phase is a relatively rare but serious complication that has been reported less frequently and is thought to result from the mobilization of sequestered calcium from damaged tissue. A dual-center retrospective case series reported hypercalcemia in 27 of 295 patients with rhabdomyolysis, corresponding to a prevalence of 9.2% [5]. The present case is particularly notable because of the severity and refractory nature of hypercalcemia during the recovery phase of rhabdomyolysis-associated acute kidney injury (AKI) despite conventional therapy, including low-calcium hemodialysis, underscoring the importance of careful monitoring and timely, judicious management.

## Case presentation

A 36-year-old man with a history of methicillin-resistant *Staphylococcus aureus *(MRSA) cellulitis, type 1 diabetes mellitus, alcohol use disorder, intravenous drug use, depression, and psychosis was found unresponsive on the street and brought to the hospital. On presentation, he was febrile to 101°F, clinically dehydrated, and hemodynamically unstable. The initial laboratory evaluation is shown in Table 1.

**Table 1:** Laboratory test results on admission

Test	Result	Reference Range
Serum		
Sodium	154 mmol/L	135-145 mmol/L
Serum Osmolality	354 mOsm/kg	275-295 mOsm/kg
Potassium	5.4 mmol/L	3.5-5.0 mmol/L
Blood Urea Nitrogen (BUN)	119 mg/dL	7-26 mg/dL
Creatinine	12.79 mg/dL	0.71-1.16 mg/dL
Calcium (Albumin-Adjusted)	10.02 mg/dL	8.4-10.2 mg/dL
Phosphorus	16.5 mg/dL	2.8-5.1 mg/dL
Hemoglobin	20 g/dL	13.5-17.5 g/dL (male) / 12–16 g/dL (female)
Urinalysis		
Protein	2+	Negative
Blood	3+	Negative
RBCs	21-50 /HPF	0-2/HPF
Ketones	Trace	Negative
Urine Toxicology	Positive for amphetamines	Negative
Arterial Blood Gas (ABG)		
pH	6.91	7.35-7.45
PCO₂	61 mmHg	35-45 mmHg
HCO₃⁻	12.2 mmol/L	23-30 mmol/L
Lactic Acid	12.1 mmol/L	<2 mmol/L

**Figure 1:**
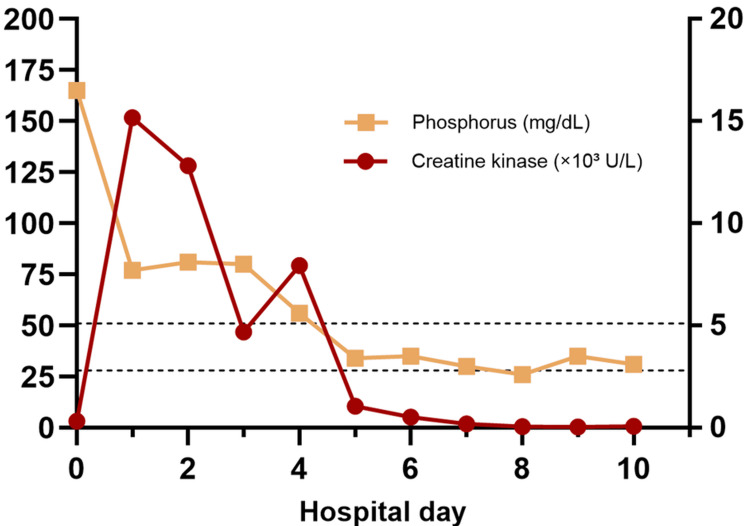
Creatine kinase and phosphorus trends during the early phase of rhabdomyolysis Peak daily creatine kinase (CK; red circles, left Y-axis) and serum phosphorus (brown squares, right Y-axis) are shown across the initial hospital course. CK rose rapidly following admission, peaking at 151,690 U/L on day one. Phosphorus was concurrently elevated during the period of peak muscle injury and gradually normalized as CK levels declined below 75,000 U/L. The area within the gridlines represents the normal reference range for serum phosphorus. Image created by authors using Python and GraphPad Prism (GraphPad Software, Inc., San Diego, CA)

The patient was intubated and started on aggressive isotonic Na bicarbonate fluid resuscitation and vasopressor support. Shortly after admission to the medical intensive care unit, his potassium increased to 9.2 mmol/L and manifested severe electrocardiographic changes. He was refractory to medical therapy, prompting emergent initiation of intermittent hemodialysis (iHD). His hospital course was further complicated by *Pseudomonas *and MRSA bacteremia, septic shock, persistent respiratory failure requiring mechanical ventilation, and delayed renal recovery requiring ongoing iHD. On hospital day three, he was transitioned to continuous renal replacement therapy (CVVHD) due to hemodynamic instability. His creatine kinase (CK) level increased to a maximum of 151,960 U/L. CK levels were serially monitored through hospital day 10, at which point the level had decreased to 668 U/L, and monitoring was discontinued (Figure 1). As his hemodynamic status improved, he was transitioned back to iHD on hospital day 14.

**Figure 2:**
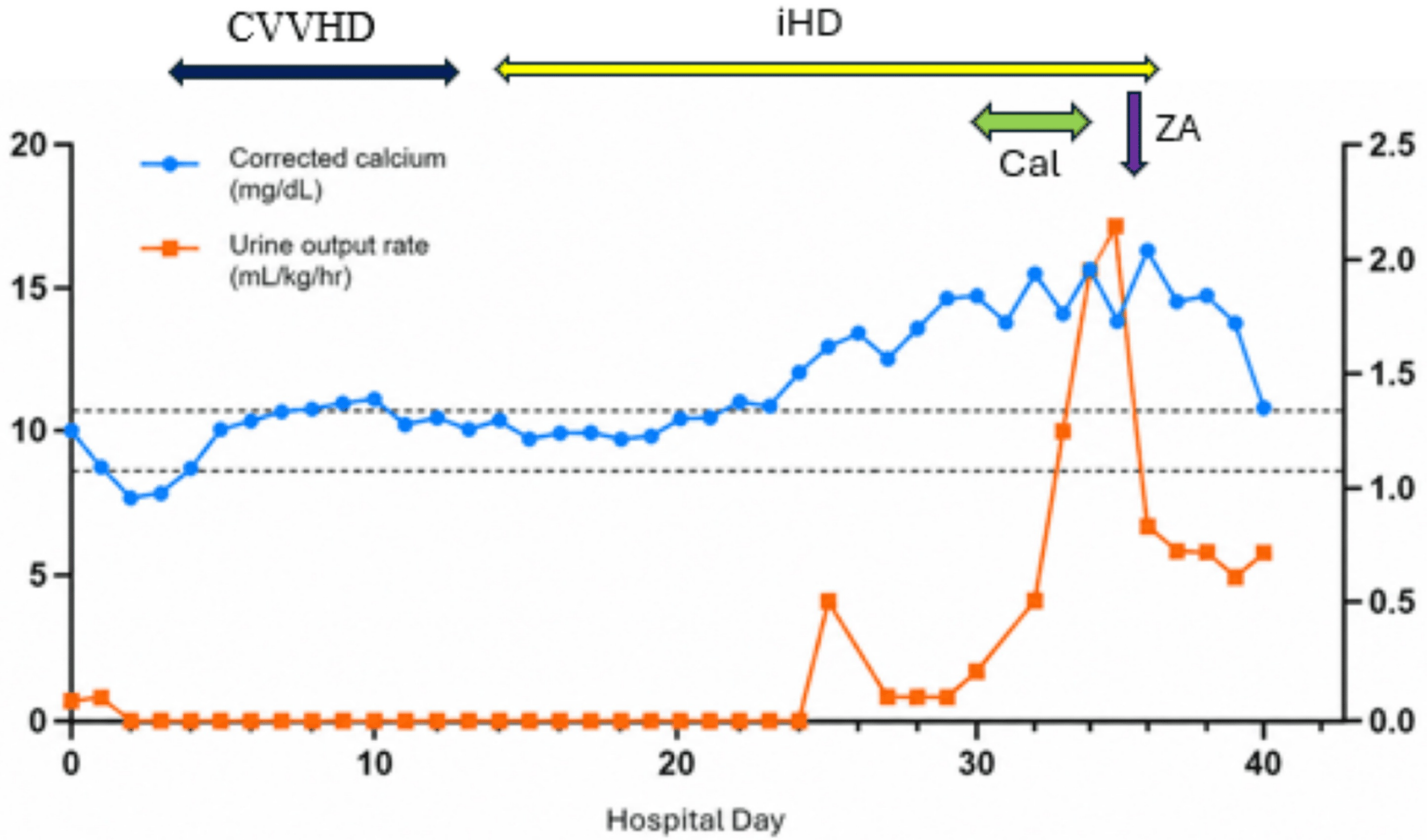
Biphasic calcium trend in rhabdomyolysis and hypercalcemia corresponding with renal recovery Albumin-corrected serum calcium (blue circles, left Y-axis) and urine output rate (orange squares, right Y-axis) are shown across the hospital course. Calcium levels were initially low during the early phase of rhabdomyolysis (hospital days 1-4). Urine output remained negligible through hospital day 24, after which it began to increase. This was concurrent with a rise in calcium that peaked at 15.96 mg/dL despite calcitonin (CAL) and dialysis therapy. A trial 4 mg dose of zoledronate (ZA) was administered on hospital day 36, with calcium trending toward the normal range in subsequent days. The area within the gridlines represents the normal reference range for serum calcium. CVVHD: continuous venovenous hemodialysis; iHD: intermittent hemodialysis Image created by authors using GraphPad Prism (GraphPad Software, Inc., San Diego, CA) and Powerpoint (Microsoft® Corp., Redmond, WA)

Early during hospitalization, the patient experienced transient hypocalcemia, which was normalized on day five (Figure 2).

**Figure 3:**
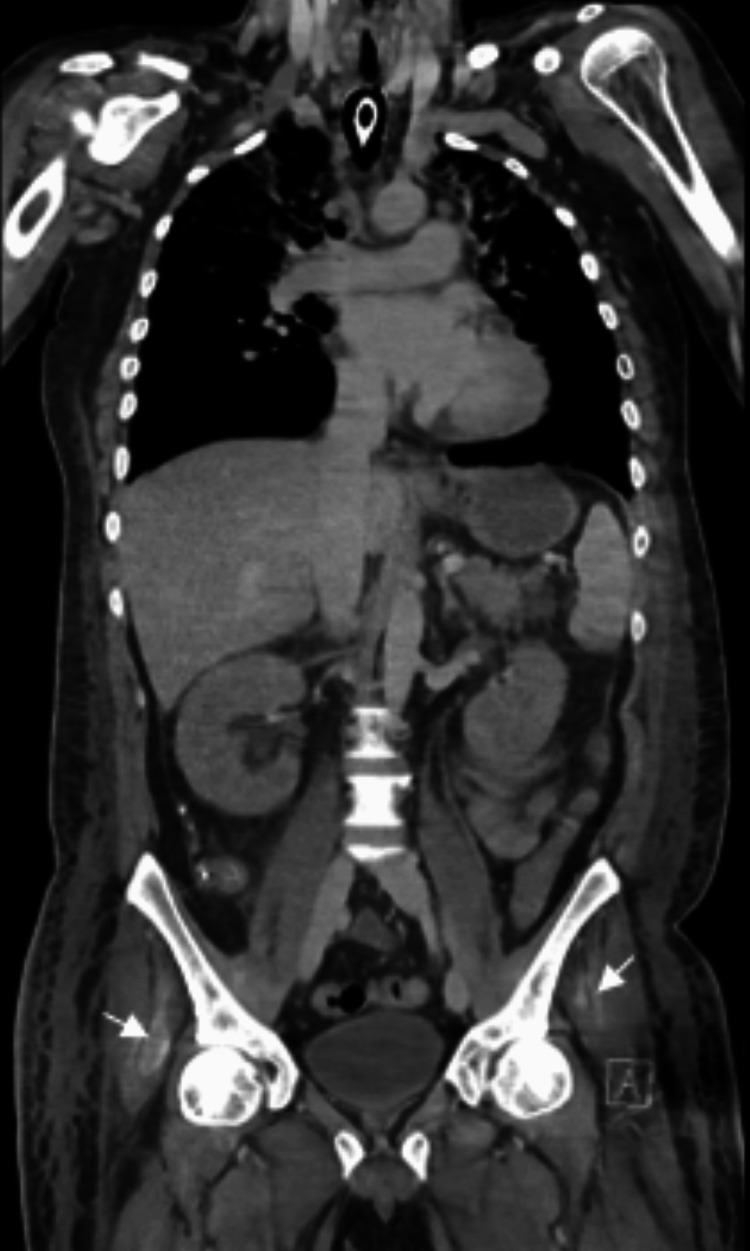
Calcium deposition in rhabdomyolysis-associated muscle injury Contrast-enhanced axial computed tomography of the abdomen and pelvis obtained on hospital day 26 demonstrates abnormal heterogeneous enhancement of the gluteal musculature (arrows).

On hospital day 25, calcium began to rise (10.5 mg/dL) as renal function gradually improved and urine output increased. Computed tomography (CT) of the abdomen and pelvis, obtained the following day (day 26), showed heterogeneous enhancement of the bilateral gluteal musculature, concerning for muscle injury-associated calcium deposition (Figure 3).

Laboratory evaluation showed a parathyroid hormone (PTH) level of < 4.0 pg/mL (reference value: 10-65 pg/mL) and a vitamin D level of 22.5 ng/mL (reference value: 20-50 ng/mL), making parathyroid-mediated and vitamin D toxicity etiologies unlikely. PTH-related peptide (PTHrP) was mildly elevated to 3.6 pmol/L (reference value: < 2.3 pmol/L). Calcium and vitamin D supplementation were discontinued, serial monitoring of ionized calcium was initiated, and iHD was continued with a dialysate calcium concentration of 2.0 mEq/L. A trial of calcitonin 400 U twice daily was initiated on hospital day 30, and the dialysis calcium bath was further reduced to 2.0 mEq/L. A daily dose of 80 mg of intravenous furosemide was also administered on hospital days 33-34 to promote urinary calcium excretion. Despite these measures, hypercalcemia persisted (Ca 15.48 mg/dL), prompting discontinuation of calcitonin therapy on hospital day 34. This occurred as the renal function continued to improve, with urine output increasing to 2.1 mL/kg/hr and serum creatinine decreasing to 1.34 mg/dL by day 35 (Figure 2). Calcium reached a peak serum concentration of 15.92 mg/dL on day 36. In view of the severe refractive hypercalcemia, a dose of zoledronate 4 mg was administered, and his response to bisphosphonate therapy was monitored in the subsequent days.

His respiratory status gradually stabilized on mechanical ventilation via tracheostomy, and he was transferred to the internal medicine floor on day 38 (Ca 14.32 mg/dL). By day 40, he was deemed clinically stable for discharge to a long-term acute care facility, with hemodialysis scheduled three times weekly and close outpatient nephrology follow-up. At discharge, his calcium had decreased to 10.28 mg/dL, and his urine output was 0.7 mL/kg/hr. Additional laboratory values included potassium of 3.6 mmol/L, bicarbonate of 25 mmol/L, blood urea nitrogen of 28 mg/dL, creatinine of 1.57 mg/dL, ionized calcium of 1.30 mmol/L, and phosphorus of 2.6 mg/dL.

In summary, the patient developed rhabdomyolysis-associated AKI manifested as early hypocalcemia, followed by refractory hypercalcemia during the renal recovery phase, necessitating careful monitoring and timely intervention.

## Discussion

Calcium disturbances in rhabdomyolysis are rare but serious complications with variable onset and severity throughout the disease course. If left untreated, they may rapidly lead to severe electrolyte disturbances, mental status deterioration, arrhythmias, organ dysfunction, and even death [6,7]. Our patient developed a biphasic calcium pattern, characterized by early transient hypocalcemia, followed by severe, refractory hypercalcemia during the recovery phase of rhabdomyolysis, reaching a peak serum calcium of 15.96 mg/dL. Such marked elevations are exceedingly rare and have been reported in a prior case [8]. This report provides insight into the clinical presentation of a biphasic calcium disturbance with refractory hypercalcemia in rhabdomyolysis, which to date remains sparsely documented in the literature.

The mechanisms underlying calcium disturbances in rhabdomyolysis are incompletely understood, although several explanations have been proposed. Hypocalcemia during the early phase is thought to result primarily from intracellular sequestration of calcium within injured myocytes. Under normal physiologic conditions, skeletal muscle cells maintain very low intracellular calcium concentrations through ATP-dependent ion transport mechanisms. During acute muscle injury, ATP depletion may disrupt these regulatory mechanisms, allowing uncontrolled calcium influx into damaged cells, resulting in calcium depletion from the extracellular space. In addition, phosphorus released from muscle breakdown may further lower systemic calcium by precipitating calcium phosphate within injured muscle tissue. As renal function improves during the recovery phase, mobilization of previously sequestered calcium from damaged tissues may lead to rebound hypercalcemia [8].

Our patient first developed hypocalcemia (8.4 mg/dL) on day one, as shown in Figure 2. At that time, he was managed with IV calcium supplementation and iHD, and serum calcium normalized by the end of day four (9.44 mg/dL). Although this suggests a relatively brief hypocalcemic phase, the patient was transitioned to CVVHD from days 3-14 due to hemodynamic instability. This raises uncertainty about the true duration and severity of hypocalcemia, as continuous dialysis may have masked ongoing hypocalcemia beyond what was readily apparent in routine laboratory studies.

Hypocalcemia is a common early feature in rhabdomyolysis; however, its onset, duration, and contributing factors remain incompletely characterized, with limited data beyond a case series suggesting more pronounced hypocalcemia in patients with coexisting AKI. In milder cases, hypocalcemia is typically transient and self-limited, with prior reports describing normalization of calcium levels generally within four to six days of presentation [9-11]. This is consistent with the timeframe observed in our patient, albeit potentially masked by CVVHD.

This early hypocalcemia is consistent with a biphasic disturbance in calcium homeostasis, which later became evident as hypercalcemia during the recovery phase. That is, following an interval during which serum calcium remained within the normal range, calcium began to rise on day 25 (11.46 mg/dL), as shown in Figure 2. This increase coincided with early signs of renal recovery, as evidenced by improving urine output on that same day (0.5 mL/kg/hr).

This biphasic process, particularly when characterized by a prolonged interval between the hypocalcemic and hypercalcemic phases, carries important clinical implications. Thus, interval calcium measurements obtained during the clinical course of rhabdomyolysis may appear normal despite ongoing, dynamic calcium redistribution or, as in our case, be potentially confounded by CVVHD. These fluctuations may be further influenced by coexisting factors, such as reperfusion injury in trauma-associated rhabdomyolysis, resuscitation with citrate-containing blood products, or immobilization [12-14]. As a result, calcium trends in rhabdomyolysis are highly variable across patients and underlying etiologies, particularly during the hypercalcemic phase. For instance, prior reports describe the onset of hypercalcemia most commonly between two and four weeks after the episodes of rhabdomyolysis; however, cases have been documented as late as six weeks after the initial presentation [7,15]. These observations highlight the importance of continued serial calcium monitoring even after apparent correction of early abnormalities during the hospital course, with interpretation of results within the broader clinical context. Conversely, the absence of early calcium disturbances should not preclude consideration of rhabdomyolysis when clinical suspicion remains high.

Once hypercalcemia was identified, a thorough evaluation to exclude alternative etiologies was undertaken, proceeding systematically from the most common causes to less obvious possibilities. In our patient, prolonged immobilization raised concern for immobilization-associated hypercalcemia, a relatively common complication in acutely ill patients with extended hospitalizations. However, CT imaging obtained during hospitalization demonstrated heterogeneous enhancement within the gluteal musculature, suggesting ongoing calcium mobilization from injured muscle as the more likely etiology (Figure 3). As shown in other studies, bone scintigraphy may also demonstrate increased radiolabeled phosphate uptake in the same regions where enhancement is identified on CT imaging, supporting the presence of calcium deposits in injured muscle [2,6,16].

Although hypercalcemia of malignancy was considered unlikely, PTHrP was obtained to further exclude this possibility. The elevated result, however, should be interpreted cautiously in the context of renal failure. PTHrP is physiologically cleaved into N-terminal and C-terminal fragments, and laboratory assays exist for both of them. While both fragments may be elevated in malignancy-associated hypercalcemia, studies have shown that the C-terminal fragment, owing to its longer half-life, can accumulate in chronic kidney disease and renal failure, leading to falsely elevated levels despite the absence of malignancy. In contrast, N-terminal PTHrP is less dependent on renal clearance and therefore represents a more reliable diagnostic assay in this context [17,18]. Because most laboratories default to measuring C-terminal PTHrP, an isolated elevation in patients with renal failure is nonspecific and should ideally prompt confirmatory testing with an N-terminal PTHrP assay or repeat testing after recovery of renal function. Finally, concern for rare endocrine etiologies of hypercalcemia, including those associated with excess insulin-like growth factor-1 or adrenocorticotropic hormone, may warrant evaluation in consultation with endocrinology [6].

The cornerstone of initial management of hypercalcemia in rhabdomyolysis is aggressive intravenous hydration, loop diuretics, and hemodialysis in the event of persistent hypercalcemia. However, when osteoclast activity is increased, calcitonin and bisphosphonates may be considered. In our case, calcitonin was administered for four days (Figure 2) in combination with loop diuretics and intermittent hemodialysis with a reduced-calcium dialysate. After an inadequate response and worsening hypercalcemia (15.96 mg/dL), a single 4 mg dose of zoledronate was given on day 36, with near-normalization of serum calcium observed approximately four days later.

The use of bisphosphonates in this setting has been rarely described, and there are currently no established guidelines for their use in rhabdomyolysis-associated hypercalcemia. To our knowledge, only a few cases have described successful treatment of dialysis-resistant hypercalcemia in rhabdomyolysis using intravenous pamidronate and zoledronate [16,19,20]. Notably, both our case and at least one prior report demonstrate an improvement in calcium within approximately four to five days following bisphosphonate administration, consistent with the expected time to peak pharmacologic effect [20].

Although these observations suggest a potentially useful therapeutic role for bisphosphonates in rhabdomyolysis-associated hypercalcemia, the precise mechanism remains unknown. Bisphosphonates bind to hydroxyapatite crystals in bone and inhibit osteoclast-mediated bone resorption, thereby suppressing calcium release from bone turnover. This mechanism may indirectly help offset the excess calcium released during the recovery phase of rhabdomyolysis [5,20]. However, it remains unclear whether bisphosphonates have a direct effect on the calcium deposits within the injured muscle itself. Future studies could further characterize this using radiolabeled bisphosphonates in biopsied injured muscle tissue [21-23]. In any case, caution should be exercised when using bisphosphonates in patients with renal impairment, as these agents carry a risk of nephrotoxicity and may require a dose adjustment.

Moreover, assessment of osteoclast-mediated bone resorption with serum markers is limited in kidney failure, as most of them depend on renal excretion and accumulate with reduced glomerular filtration rate (GFR). Tartrate-resistant-5 acid phosphatase (TRAP-5) is independent of GFR and may be more reliable in AKI; it was not available in this case [24].

In addition to pharmacologic treatment of hypercalcemia, management should also address potential contributing factors, such as immobilization and nutritional status. Our patient remained intubated and bedridden for a significant portion of his hospital course, precluding early mobilization with physical therapy.

Management of coexisting electrolyte disturbances in rhabdomyolysis is also important. Severe hyperkalemia, a potentially life-threatening complication, is often treated with intravenous calcium (gluconate or chloride) to stabilize the cardiac membrane. Although appropriate, this intervention potentially worsens hypercalcemia, particularly during the recovery phase of rhabdomyolysis. This underscores the importance of close calcium monitoring when administering calcium-containing medications or fluids in affected patients. Additionally, even after hypercalcemia has been resolved, close follow-up is warranted to monitor calcium homeostasis and muscle recovery, evaluate potential medication-related adverse effects, and assess renal recovery. At a minimum, follow-up testing should include serum creatinine, calcium, phosphorus, and albumin; repeat parathyroid hormone and vitamin D levels; and reassessment of equivocal laboratory findings during hospitalization, such as the elevated PTHrP observed in our patient.

Finally, from an operational standpoint, dialysate with a sufficiently low calcium concentration was not readily available at our hospital to further control hypercalcemia. In perspective, a previous case report that used calcium-free dialysate in a patient with rhabdomyolysis achieved improvement in serum calcium from 15.1 to 7.3 mg/dL [8]. This highlights a critical logistical aspect for nephrology services: maintaining ready access to the otherwise rarely used, low-calcium dialysates, particularly during periods with a higher incidence of rhabdomyolysis, such as the hot, dry summer months and in natural disasters.

## Conclusions

Rhabdomyolysis can cause complex disturbances in calcium homeostasis during both the acute and recovery phases of AKI. Severe delayed hypercalcemia in rhabdomyolysis-associated AKI is a rare but important complication that may persist despite standard therapy. In this case, hypercalcemia improved with calcitonin, zoledronic acid, dialysis support, and eventual renal recovery; however, conclusions regarding treatment efficacy should be interpreted with caution, given the single-case nature of this report. Bisphosphonates may be considered selectively in refractory cases, while close biochemical monitoring and individualized management remain essential during recovery.
